# The Hospital

**Published:** 1918-01-12

**Authors:** 


THE
### INSTITUTIONAL WORKER
**Being a Special Supplement to "The Hospital"**

### OUR BUREAU OF INFORMATION.

### Rules for Correspondents.

**1. Every letter must be accompanied by the coupon to be cut from the back cover (inside page) of THE HOSPITAL, current issue, and must contain the name and address of the correspondent with pseudonym for publication if desired. Replies by post cannot be given save under exceptional circumstances at the Editor's discretion.**

**2. Letters from Approved Homes in reply to special needs published in the Bureau should state terms and full particulars, and be sent prepaid under cover to the Editor of the Bureau with name written across coupon for identification.**

**3. Proprietors of Homes which have not yet been entered on the List of Approved Homes, but have spare accommodation likely to suit special needs, are invited to write for an application form for registration. The fee for registration, which includes two announcements of the Home in the Bureau and other privileges, is 10s.**

**4. All communications to be addressed to the Editor of THE HOSPITAL, 28 Southampton Street, Strand, London, W.C. 2, and marked "Bureau of Information."**

### INSTITUTION FACTS AND FIGURES.

### The Equipment of an Out-Patient Department

#### Answer to November Question.

**By M. K. S.**

**THE question was :**

**State what you consider necessary for the general equipment of a new Out-Patient Department and Accident Room, and the procedure to be adopted for the provision of such requirements.**

As equipment only is asked for, we therefore understand that the fabric and building, with fittings, is already in existence. This broadly comprises a dispensary, waiting-hall, surgery, and surgeons' rooms, and special rooms for different departments. The out-patient department should be attached to the main building, but need not necessarily be too near to it. A clerk's office will be arranged near the entrance, and a tea bar, where patients coming from a distance can obtain simple food, should also be in a central position. The latter will be found to more than pay its way, and is easily managed and run.

There should also be ample lavatory accommodation, sinks, wash-basins, etc. The out-patient department should be connected by telephone to the main building, and easily accessible to the lift there should be carrying chairs and stretchers always at hand for the removal of patients to the wards.

The *x*-ray department is usually under the same roof, and the working of this special department comes either directly under the supervision of the out-patient staff or the massage staff.

In a busy and congested district this department will always be "on duty" for accidents, the special departments having special times and days for their own cases.

The venereal clinic in most cases is also attached to this department and worked from it.

THE WAITING-HALL.

This should be a large, well-lighted, airy building, heated, and as far as possible free from draughts.

It should be furnished with a clock, forms with backs, direction boards to assist the patients where to attend, fire apparatus, sand-buckets, etc.

New and old patients should have special seating, the hatch for medicine (dispensary) should when possible lead to the exit of the department, so that the patients, after obtaining their medicine, can leave the hall without returning and causing confusion.

Silence should at all times be the rule in the waiting-hall.

THE SURGERY.

In this department all the daily dressings and minor operations are as a rule attended to. Opening from it should be a surgeon's room for the examination of patients, and a recovery room for those patients who have had an anæsthetic or are waiting to be discharged, not being suitable for admission to the wards.

These three rooms would therefore be directly under the supervision of the sister. In London, and probably in the larger provincial towns, a ward is attached to the out-patient department where accident cases too ill or too slight to admit to the wards are nursed.

This ward or wards has male and female beds. The cases usually remain for twenty-four hours and are purely casualty cases. These wards are not required in the smaller provincial hospitals—the pressure is not so acute, nor is the average of accidents so large. Of course, now in the days of air-raids, especially in those areas where these raids most frequently occur, such a provision has been thought of and partially arranged for. Happily the civil hospitals have not been called upon to a great extent to admit large numbers of severe casualties so far from these raids.

The equipment of the wards is similar in every respect to the ordinary wards of the hospital.

The surgery should be a large room with a good light, heated but not overheated. There should be an excellent supply of hot and cold water, good sinks, a large steriliser, plenty of boiling water, fixed slabs or tables for the utensils required for dressings, one or two couches, an operation table with fixtures, rounders to hold soiled dressings, etc., a locked instrument cupboard, a similar cupboard to contain stimulants, poisons, and other drugs used in the surgery, instruments for tracheotomy, stomach wash-out, tourniquet, all splints for first-aid, a wagon to hold lotions, drums to contain dressings of every description, and boxes to hold bandages, slings, etc. All these articles should be so arranged that they are ready to hand.

The secretarial work involved, for which the sister is in part responsible, is best attended to in one of the rooms off the surgery.

Screens with washing covers, blankets, pillows with mackintosh covers, chairs, a long cupboard with a stock of splints, appliances, and linen, must also be in the close vicinity.

In London hospitals and some provincial hospitals it is usually the custom to ring a casualty bell which is attached from the outer gate to the surgery. In this way the staff are in part prepared for the case before it arrives, and can arrange accordingly.

Attached to the out-patient hall will be the different departments which treat special cases other than accidents or daily dressings.

These will be the ophthalmic department, the medical department, the women's diseases department, skin, ear and nose, the dental department, the massage department, and the

(*Continued on page* 2, *col.* 3.)

### JANUARY QUESTIONS.

**1. Extending a Hospital Engineer's Work.**

In what ways can a hospital engineer be useful in addition to his routine work of attending to the heating apparatus?

**2. A Domestic Matter in a Nurses' Home.**

Describe the cleanest, quickest, and most careful method of washing and drying crockery in a nurses' home to accommodate 180 persons.

### RULES.

The following rules must be observed:—

1. Contributions must be written on one side of the paper. Brevity and terseness are desirable features. The MSS. must bear the name and address of the sender and be accompanied by coupon to be cut from the back cover (inside page) of the current issue of The Hospital. A pseudonym must be chosen if the name is not to be published.
2. Contributions must be addressed to the Editor of The Hospital, Institutional Worker Supplement, 28 & 29 Southampton Street, Strand, London, W.C. 2, should reach him before the end of the current month, and be marked in the left-hand corner "Facts and Figures."

**A minimum payment of Five Shillings will be made for each published answer to the January Questions.**

### QUESTIONS INVITED.

In connection with our Question Box, we offer every month five shillings each for the two best questions which are sent in for consideration. The questions may be on any institutional subject and concern any department, from the secretary's to the porter's, or the matron's to the domestic staff; the one essential is that they must be practical and deal with points that have a definite relation to hospital or institutional administration, upkeep, finance, or management. Points relating to artificers' work, housekeeping, the laundry, out-patients, and other practical matters will be welcome. It is hoped that thus, when difficult or doubtful points arise in the course of their work, institutional workers will be encouraged to put them in the form of a question and send them to the Editor, The Hospital Bureau of Information, 28 Southampton Street, Strand, W.C. 2, marked "Question Box."

The best questions will be published in due course and our readers will be given the opportunity of answering them. Thus all institutional workers may be able to co-operate with the view of helping the work and smoothing the difficulties of each other. In short, we want the Question Box of the *Institutional Worker* to be freely used by every worker habitually.

### ENQUIRIES AND ANSWERS.

### *EMPLOYMENT AND TRAINING.*

**The Almoner's Work.**

If you wish to know something of the work of a hospital almoner before deciding upon a course of training, we suggest that you read an interesting pamphlet by Miss A. E. Cummins, Lady Almoner of St. Thomas's Hospital. It may be obtained of The Scientific Press, Ltd., 28 and 29 Southampton Street, Strand, London, W.C. 2. The price is 7d. post free.—T. A. T.

**Training at a Sanatorium for Consumption.**

You might possibly be received at the Royal National Sanatorium for Consumption and Diseases of the Chest, Bournemouth. Applicants are received for one year's training from the age of eighteen, but preference is given to those who intend afterwards to enter a general hospital for complete training. No premium is required. Full indoor uniform is provided for probationers. Application should be made to the Matron.—Isa.

**Growing of Medicinal Herbs.**

Medicinal Herbs could undoubtedly be grown in the grounds of an institution, provided the conditions were suitable, and in some parts of the country this form of horticulture has been taken up by private individuals. It is difficult, however, to say whether any financial profit would accrue. We suggest that you write to The Scientific Press, Ltd., 28 and 29 Southampton Street, Strand, London, W.C. 2, for particulars of books upon the subject. —L. L.

### *MISCELLANEOUS.*

**Sterilisation of Rubber Gloves.**

It should be remembered that whatever method of sterilisation be employed, the effect is always injurious. Tests show that immediately after sterilisation by some methods rubber gloves are not so firm as before, and they have a somewhat swollen, white appearance. This, however, is no indication of permanent damage, for if allowed to rest for some time they will recover their form and quality to a large extent. This fact should be borne in mind. Much saving can be effected by allowing some hours to elapse between sterilisation and use.—K. A.

**The Object of Appointing an Almoner.**

While your purpose to appoint an inquiry officer who would see that your hospital is not abused by persons able to afford a doctor's fee is good so far as it goes, the payment of a special officer to weed out these cases would scarcely be justified by the result. You would be well advised to go a step further. You need to seek out those cases requiring extraneous aid, and find for them that assistance which will enable them to derive the fullest benefit of the medical or surgical treatment given. The after-care of patients is of extreme importance, and money is well spent in this direction. The services of a trained almoner could not be obtained for less than £120 a year, though doubtless you may find a lady with experience of social work, or with a special capacity for it, who would be willing to accept a nominal salary.—A. C.

**The Value of the Out-patient Department.**

We are of opinion that you underrate the value of the out-patient department. You should remember that although the majority of cases are trivial and that the few serious ones feed the in-patient department, which is already over-burdened, on the other hand the out-patient department is the place of preventive medicine. It is here that disease is recognised in the incipient stages and treated before the course of the disease necessitates the patient taking to his bed. It is obvious, therefore, that the out-patient department, whilst it feeds the in-patient department, at the same time reduces the demands upon its accommodation.—Drastic Measures.

### THE EQUIPMENT OF AN OUT-PATIENT DEPARTMENT.

(*Continued from page 1.*)

orthopædic. Each must be complete in detail and under the supervision of the sister and her staff. To provide the equipment of these departments, should the building be absolutely new, a visit to other hospitals, preferably those which are up to date and running smoothly, would assist considerably.

The large instrument-makers, who supply everything required for such a department, are willing and able to advise and recommend what directly applies to them, but there are many other details which only practical experience can give real assistance in choosing and arranging. A few of these which may be mentioned in the limited space are (1) the flooring, (2) the lighting and ventilation; (3) the lavatory and sink arrangements; (4) the individual running successfully of such a department. It appears to be an excellent plan to attach the isolation department, either directly or indirectly, to the out-patient department building, and close at hand should be the mortuary chapel and *post-mortem* rooms and the furnaces where waste is destroyed.

